# Audio signal analysis using a modified forward–forward algorithm with enhanced segmentation for soil pest detection

**DOI:** 10.1038/s41598-025-15770-7

**Published:** 2025-08-27

**Authors:** Tusar Kanti Dash, Anurag Raj, Satyajit Mahapatra, Ganapati Panda

**Affiliations:** 1 Electronics and Communication Engineering, C V Raman Global University, Bhubaneswar, 752054 India; 2 Computer Science and Information Technology Department, C V Raman Global University, Bhubaneswar, Odisha 752054 India; 3 Department of Information and Communication Technology, Manipal Institute of Technology, Manipal Academy of Higher Education, Manipal, 576104 India

**Keywords:** Pest detection, Smart agriculture, Forward–backward algorithm, ASR, STE, Audio signal processing, AI, ML, Computational science, Electrical and electronic engineering

## Abstract

The presence of pests in soil costs the agriculture industry billions of dollars every year since it reduces crop yields and raises preventive costs. The pest detection in soil is vital for maintaining healthy crops, optimizing pest management, and ensuring economic and ecological sustainability. There are several invasive and non-invasive methods available for pest detection, where invasive methods are costly as well as time-consuming compared to the non-invasive methods. From various non-invasive methods, audio-based pest detection in the soil is one of the effective, low-cost tools. The generation of pest sounds is random in nature and contains a lot of inactive and background noisy portions in the recorded sound signals. To reduce the unnecessary computations in analyzing the inactive portions, an improved audio activity detection algorithm has been designed in this paper using Short Time Energy features for segmentation, which provides an average of 20% less computational requirements as compared to the baseline models. In the second step, the Forward Forward Algorithm has been used for its benefits in enhanced numerical stability, simplified computations, and enhanced precision over traditional back propagation-based algorithms. For improved performance in the detection of pests in soil, the traditional FF algorithm has been further updated by using root mean square in the goodness and loss function calculation. Through the comparative analysis with several baseline models, it has been observed that the proposed method consistently provides an average of 5% enhanced performance.

## Introduction

Monitoring pests in the soil is a critical part of integrated pest management, especially as climate change and rising food demands put added challenges on agriculture^1^. Early detection helps farmers avoid big crop losses and reduce pesticide use^2^. Soil pests contribute to significant global crop losses with an estimated 20–40% loss annually, amounting to over $220 billion worldwide. Practices like crop rotation, cover cropping, and reduced tillage improve soil health by encouraging helpful microbes that naturally suppress pests^3,4^. New tools like audio and Internet of Things (IoT)-based systems are making pest detection easier and more sustainable^5–7^. With Artificial Intelligence (AI), their accuracy has been increasing^8^. However, background noise is challenging^9^, and these tools work continuously without disturbing the soil^10–13^. Automatic Speech Recognition (ASR) also helps speed up detection. It has been observed that models like You Only Look Once version 5 (YOLOv5) have shown good results for spotting pests in images^14^, and Support Vector Machines (SVM) are better for sound-based tasks^15^. Deep learning tools like YOLOv5s and Residual Network (ResNet) are powerful but need strong internet and energy, which can be tough in rural areas^16^. The deep learning models need powerful hardware, fast internet, and a lot of memory that are hard to come by in rural areas.

**Table 1:** Comparative analysis between forward–forward and backpropagation algorithms.

Parameter	Forward–forward	Backpropagation
Output	Doesn’t have a dedicated output layer	Have a dedicated output layer
Learning process^38^	Employs two forward passes per layer (one with positive data and one with negative data), with each layer learning independently based on local loss functions	Involves a forward pass to compute activations and a backward pass to propagate errors and update weights across all layers
Computational efficiency^38^	Potentially offers more parallelization since each layer operates independently, though current implementations have shown it to be slower than backpropagation	Can be computationally intensive due to the need for gradient calculations and sequential weight updates
Biological plausibility^44^	Designed to be more aligned with biological processes by eliminating the need for a backward pass, making it the subject of interest in neuroscience-inspired AI research	Often criticized for lacking biological realism, as the backward pass does not have a clear analog in biological neural networks
Gradient issues	Less susceptible to vanishing gradients	Prone to vanishing/exploding gradients
Memory requirements	Lower (no need to store for backward pass)	Higher (must store activations and gradients)

Audio monitoring is a low-cost as well as non-invasive method for pest detection. It works well with techniques like Fast Fourier Transform (FFT) and Linear Predictive Coding (LPC) with higher accuracy^17,18^. Models like Convolutional Neural Network (CNN) and Bidirectional Long Short-Term Memory (BiLSTM) handle background noise better^9^. Traditional models such as Random Forest and eXtreme Gradient Boosting (XGBoost) are also useful, especially with embedded noise filtering and microcontroller support^19,20^. Soil-based sound sensors linked to smartphones alert farmers in real time^17,21^. This data also helps governments plan better and gives farmers access to eco-friendly markets^21^. For broader use, these systems need to be affordable, easy to use, solar-powered, and supported by local training. Even though Automation raises concerns about job loss^22^, it helps the farmers immensely if workers are trained^21,22^.

Traditional soil pest detection methods like digging, soil sampling, and baiting are effective but often time-consuming and physically demanding^23^. They are not very practical for implementation on large farms. They depend on expert judgment, and can catch pests in the later stage when the damage has already been done. In rural areas, doing this frequently is tough and expensive. There’s a growing demand for smarter, non-invasive solutions that can detect pests in real time with less effort. Unlike aerial imaging, which requires a clear line of sight, and subterranean methods, which often involve digging or disturbing the soil, audio-based monitoring is a non-invasive method that doesn’t harm the environment, and is easier to scale across large areas, and also cost-effective^24^. Newer tools like PepperNet use multimodal learning by combining images and text for accurate pest detection^25^. Microphone placement matters to reduce interference from wind or plants^26,27^, and syncing audio with visuals improves results^28^. Lightweight models like Mobile Networks (MobileNets) run efficiently on low-power farm devices^29^, while tools like smart traps and electronic noses cut chemical use^30^. AI-driven data fusion pulls together audio, image, sensor, drone, and weather data for smarter pest control^31–33^. YOLOv5 performs well^34^, and another model, TinySegformer, uses Transformer learning for edge-device detection^35^. Accuracy plays a crucial role in pest detection to stop waste resources^36,37^. There is a necessity for parallel computing in agriculture to handle large datasets and improve the accuracy^38^. Techniques like dropout, early stopping, and regularization prevent overfitting^39,40^, and keeping bias and variance low ensures consistent results^41^. Streamlined models and distributed sensor networks with deep learning boost performance in real-time field use^42,43^. A well-planned plantation schedule boosts yield with minimal resources by using a rule-based fuzzy method to predict optimal sowing timing from environmental data^6^.

The forward–forward (FF) algorithm is a technique developed relatively recently as an improvement over standard backpropagation by Geoffrey Hinton^44^. This method stresses a less complex and more biological way of learning and is meant to avoid some of the shortcomings of backpropagation, including computational cost and biological realism. The FF algorithm works in a manner such that it undertakes two forward passes through the network. The first pass is the ‘positive pass,’ in which the network is presented with actual samples, whereas the second pass is the ‘negative pass,’ and here the network is presented with faked or modified samples. The network is then trained to differentiate between these two passes with the aim of obtaining greater activation of the output for real data and lower activation of the output for altered data^45^. The FF algorithm makes the training process easier because there is no requirement for backpropagation, as this can be time-consuming if many computations and propagation of gradients are involved. This may have the potential to shorten the training time, especially when developing complex networks or when the hardware used has small capabilities in computation^46^. With regard to the idea of increasing the gap in activation level between the real and synthetic data, the FF algorithm can greatly help in better generalization. This could help introduce neural networks in a greater number of applications that were not previously used. Recently FF algorithm has been used for audio-spoken digit identification^47^, audio recognition^46^, ECG signal analysis^48^, and separation index maximizing^49^. Due to the real-time analysis of soil sounds in the agricultural fields, low memory-based processors are used, which require low computational complexity-based algorithms^50^.

It has been observed from the brief literature review that proper pest control will help in the protection of crops, lower the usage of chemical pesticides, and enhance sustainable agriculture practices. Through audio signal analysis, non-invasive early pest detection is possible. By using acoustic analysis for early pest detection, smart farming becomes more efficient, affordable, and precise. Recently, some related work has been reported in this direction for audio-based pest detection in agriculture. However, very few works have been reported in soil pest detection. Additionally, in most cases, deep learning methods are used, which have higher computational costs where backpropagation of error plays a crucial role. Recently, a novel FF algorithm has been proposed with simplified Implementation and a lack of need for complex gradient computation, biological plausibility, reduced computational complexity, and better generalization. However, to the best of knowledge available to the authors, these concepts of soil pest monitoring with audio signal analysis and FF algorithm have never been implemented. Additionally, no attempt has been made to modify the basic preprocessing or segmentation requirements for the analysis of audio signals for soil pest detection. Because the insects create sound randomly, analyzing the full duration of the signal requires higher computation. This work has been considered in the paper with the following research objectives.

- Development of an enhanced audio segmentation technique for faster processing and extraction of relevant audio features.
- Development of a Modified FF Algorithm for soil pest monitoring by analyzing the basic FF Algorithm for audio signal processing, with a focus on audio activity detection, signal pre-processing, and customized loss functions for improved soil pest monitoring.
- Assessment of the performance of the proposed model along with comparative analysis with base models in terms of accuracy and computational complexity.
- Validation of the proposed model’s generalization ability using multiple datasets and varying noise conditions.

The paper is divided into four sections, the first of which covers the introduction, literature review, goals of the study, and reasons for conducting it. Sect. “Materials and methods” covers the specifics of the materials and techniques used. An analysis of the findings and contributions in terms of research findings is included in Sect. “Result analysis”. Sect. “Conclusion” presents the research findings, limitations, and scope for further studies.

## Materials and methods

**Fig. 1:**
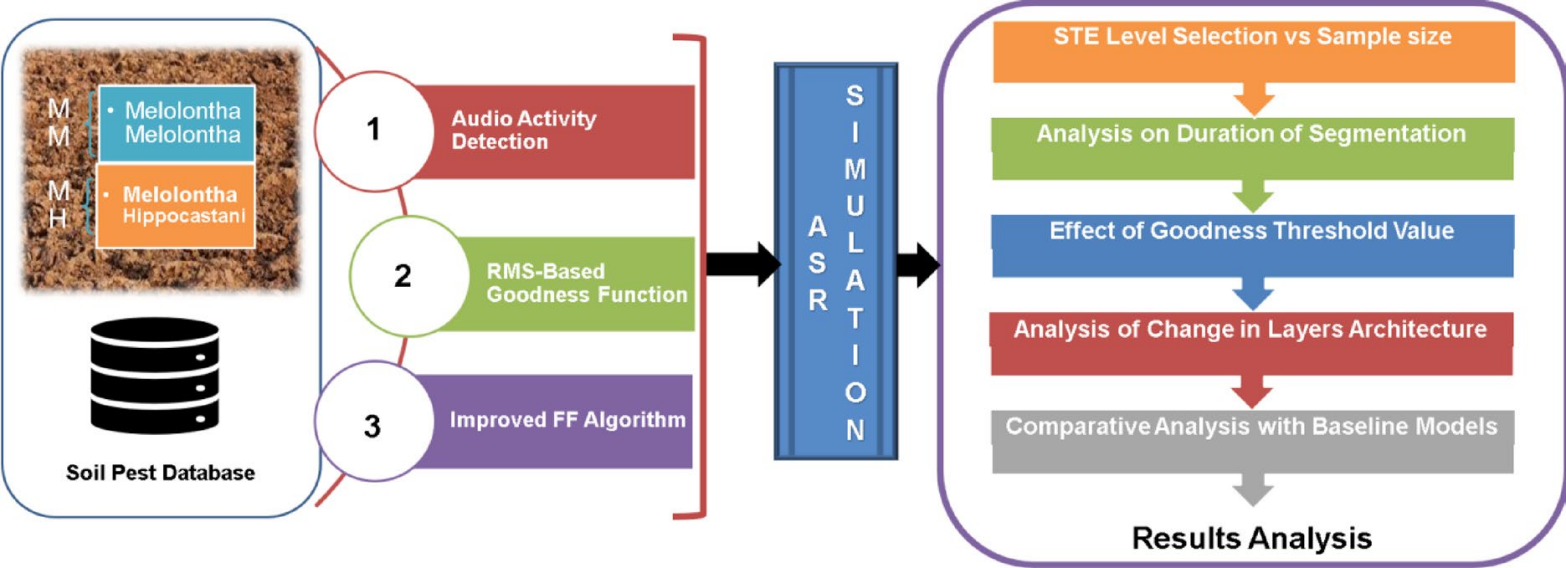
Block diagram of the proposed approach.

The block diagram of the proposed approach and its implementation steps are shown in Fig. 1. The implementation starts with database collection, followed by audio activity detection-based segmentation. The segmented audio signals are used with a modified FF algorithm having an Root mean square (RMS)-based Goodness function. The simulation-based analysis and results are analyzed from the context of Short Time Energy (STE) level selection, segmentation duration, the effect of the Goodness function, change in architecture of layers, and comparison with baseline models. The stepwise methodology for the implementation is presented in the subsequent sub-sections.

### Database used

The audio dataset used in the proposed implementation has been collected from the lab experiments done in^51^. Here, the sound of larvae of the root-feeding scarabaeidae family has been analyzed, which can be a major hazard to forest and agricultural ecosystems, and yet much about their ecology is still unclear. An acoustic data analysis technique based on the active sound generation of larvae (i.e., stridulations) has been used. The earliest known stridulation recordings for each species were obtained in a laboratory investigation using third instar larvae of the Common Cockchafer (Melolontha melolontha) (n = 38) and the Forest Cockchafer (M. hippocastani) (n = 15) housed in soil-filled containers. The larvae were acoustically monitored for five minutes apiece, and the dataset has been collected from the experimentation done in^51^. Audio recordings were captured using a TASCAM DR-05 recorder paired with piezoelectric transducers at a 44.1 kHz sampling rate in WAV format. To clean the data, the recordings were bandpass filtered between 200 and 5000 Hz—frequencies known to contain relevant stridulation signals from scarab beetle larvae while excluding background noise. The waveforms were then normalized to a peak amplitude of − 1.0 dB, and any DC offset introduced by the recording equipment was removed to center the signals around zero^51^. Each 5-min samples are further divided into samples of 10-s duration using segmentation. A total of 1400 samples have been created, combining two classes named MH and MM. The MH denotes Melolontha hippocastani, while MM denotes Melolontha melolontha classes in the species of scarab beetle and have an equal number of samples in each class.

### STE level selection for segmentation and features

In order to reduce processing time and extract relevant information, the speech processing programs distinguish between voice-active speech intervals and silent parts. STE is widely used in audio signal processing to track changes in signal intensity over short time windows. This makes it especially valuable for segmenting audio by identifying transitions between silence, speech, and other sound events. STE has been used in deep learning techniques to accurately detect speech boundaries for practical effectiveness in real-world applications^52^. Using STE and tracking how energy changes over time reveals patterns that other features might miss. In another study, STE is applied to separate speech from music, demonstrating its usefulness in classifying different audio types^53^. Consequently, voice-over-Internet-protocol algorithms, audio conferencing, speech coding, speech recognition, echo cancellation, and other speech processing applications have made extensive use of voice audio detection algorithms^54^. A similar logic has been used in the proposed method to do segmentation and subsequent selection of segments of the recorded signals, which are of an average duration of more than five minutes. Here, the STE-based segmentation has been used with the proper threshold to get more than 500 samples per class. In the proposed implementation, for STE calculation, a window size of 10 s with 0% overlapping has been used to detect the frames where STE is above a specific threshold value. Those frames are selected to form 1400 audio segments of 10 s duration each. Traditionally, a 25-ms window size with 50% overlapping is used for feature extraction. However, in the proposed implementation, as the aim is to do segmentation of long-duration signals in the first step, therefore larger window size has been taken.

### Traditional backpropagation algorithm

During backpropagation (BP)-based learning, the network goes through two distinct phases: a forward pass and a backward pass. During the forward pass, a given set of training input features is forwarded through the network, consisting of several layers, one at a time or in a batch^55^. Here, they are modified by the model parameters, i.e., the weights and biases. At each layer, activation functions such as Rectified Linear Unit (ReLU), sigmoid, tanh, etc. are applied to introduce non-linear transformations to the network. This allows the network to create more sophisticated feature representations of the input at each layer. Once the forward pass is complete, the network arrives at a prediction.

A pre-defined loss function is used to quantify the performance of the network by comparing the prediction with the actual output to obtain an error measure. The objective of the network is to minimize this loss function, resulting in the prediction being closer to the actual output. This minimization is often addressed by gradient-based methods, such as stochastic gradient descent (SGD) and its variants. During the backward pass, the loss is propagated back through the network using the chain rule of calculus, computing the gradient of the loss function with respect to the network’s parameters. This involves calculating partial derivatives layer by layer, from the output back to the input, hence the term ‘backpropagation’. The gradients are then used to update the network’s parameters, typically using a learning rate to control the size of the updates. This process, often performed in mini-batches for computational efficiency, is iterated until the error is minimal or some stopping criteria are met. Through this iterative process of forward passes and backward adjustments, the network learns to make increasingly accurate predictions on the training data. The three key points are:

- In BP, the network has a dedicated output layer that determines the network’s final prediction.
- A single, global loss function governs the entire network’s learning processes.
- The network learns the representation of the training data in such a way that for a given input, it decides the value that each of the neural units at the output layer must have in order to generate the most appropriate prediction.

**Fig. 2:**
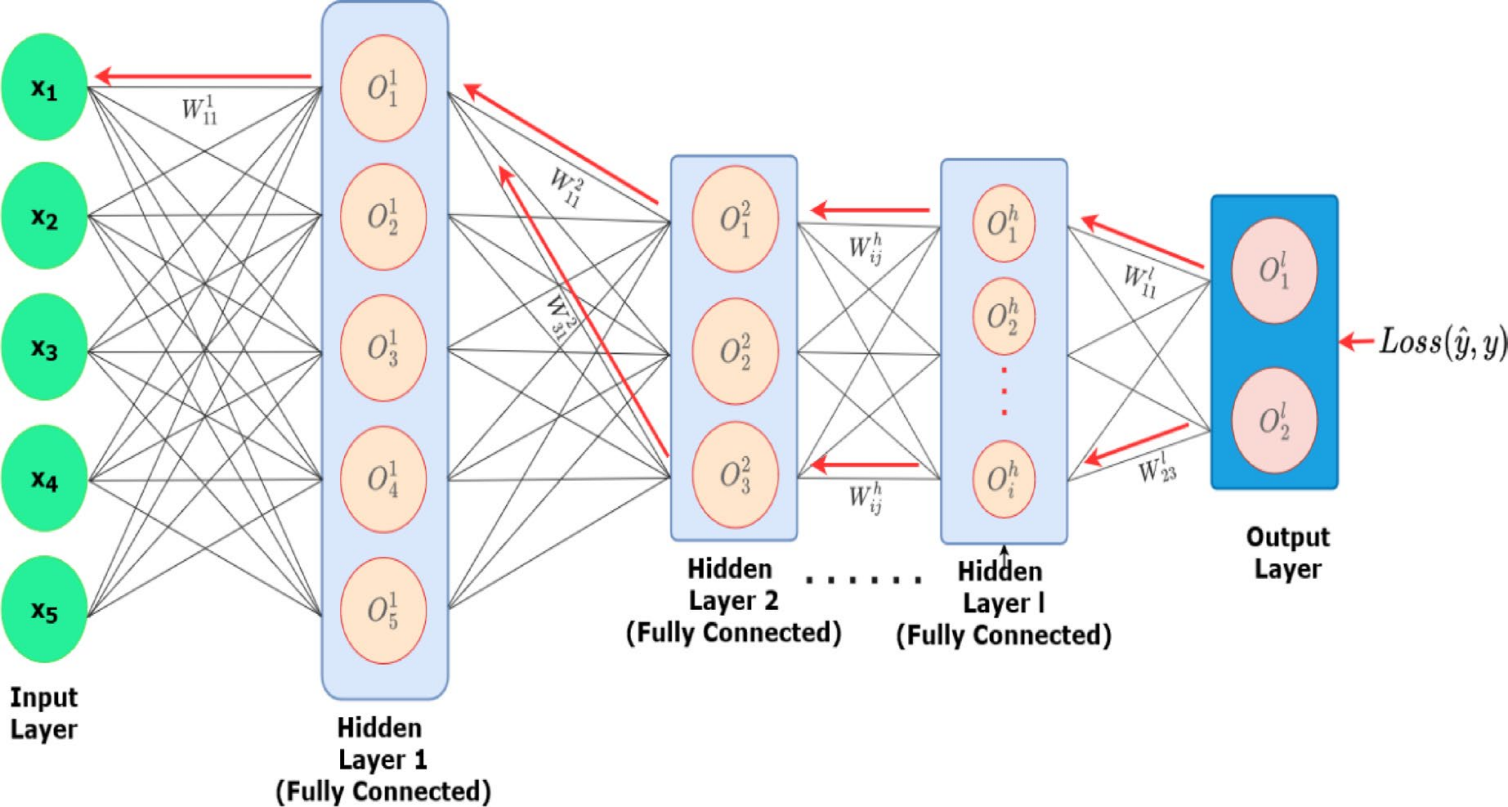
Architecture of back propagation algorithm.

The architecture of the BP algorithm is shown in Fig. 2, and the symbols used in the manuscript and their Interpretations are listed in Table 2. In the first step, the input layer (X_1_, X_2_, …) is connected to the hidden layers, and the interconnection between multiple hidden layers is shown. The forward path is shown with the connections in black color while the backpropagation path is shown in red color in Fig. 2. Considering a Neural Network* N* with multiple layers *N (N1, N2, …, Nh, …, Nl*) The Weighted Sum *S*^*h*^_i_ can be expressed as

1 $$S_{i}^{h} = \mathop \sum \limits_{j} W_{ij}^{h} O_{j}^{h - 1}$$

**Table 2:** Symbols used and their interpretations.

Symbols used	Inference
*N*	Neural network
*N*_*1*_	Number of neural units in the input layer
*N*_*h*_	Number of neural units in the *hth* layer
*N*_*l*_	Number of neural units in the *hth* layer
*n*	number of units in layer *h*
${S}_{i}^{h}$	Weighted sum
${W}_{ij}^{h}$	Weight of the connection between neural unit *i* of layer *h* and *j* of the preceding layer (*h − 1*)
${O}_{j}^{h-1}$	Generic output of a neural unit* j* of preceding layer (*h − 1*)
${S}_{i}^{h}$	Weighted sum of the outputs of the preceding layer (*h − 1*) (or the input layer) for the neural unit *i* of the layer *h*
${O}_{i}^{h}$	Generic output of neural unit *i* of the layer *h*
${\lambda }_{i}^{h}$	Activation function applied on the neural unit *i* of layer *h*
$\eta$	Learning rate (step size)
${\Delta W}_{ij}^{h}$	Derivative of ${W}_{ij}^{h}$ w.r.t the loss function
*L*	Loss function
${B}_{i}^{h}$	Error that is being propagated backwards
$\sigma :$	Logistic function
*θ:*	Threshold hyperparameter
${G}_{h}^{+}$	Probability of an input vector being classified as positive
*n:*	Number of neural units in the layer *h*
${G}_{h}^{-}$	Probability of an input vector being classified as negative
${L}_{h}$	Loss function at layer *h*
${L}_{h}^{+}$	Loss function at layer *h* for positive data
${L}_{h}^{-}$	Loss function at layer *h* for negative data
${G}_{+, i}$	*i*th element of the tensor *G*_+_
${G}_{-, i}$	*i*th element of the tensor ${G}_{-}$

The Generic output (*O*^*h*^_*i*_) of a neural unit is expressed as

2 $$O_{i}^{h} = \lambda_{i}^{h} \left( {S_{i}^{h} } \right)$$

where *λ*^*h*^_*i*_ is the activation function that has been applied to the neural unit *i* of layer *h*. The selection of a proper activation function plays a crucial role in the convergence of an ANN as it introduces non-linearity, enabling the network to learn and model complex patterns^56^. The derivatives of weight with respect to the loss function.

3 $${\Delta }W_{ij}^{h} = - \eta \frac{\partial L}{{\partial W_{ij}^{h} }} = \eta B_{i}^{h} O_{j}^{h - 1}$$

The error calculation and weight updation are done as mentioned below.

4 $$B_{i}^{h} = \frac{\partial L}{{\partial S_{i}^{h} }} = \left( {\lambda_{i}^{h} } \right){\prime} \mathop \sum \limits_{t} B_{t}^{h + 1} W_{ti}^{h + 1}$$

5 $$B_{i}^{l} = \frac{\partial L}{{\partial S_{i}^{l} }} = T_{i} - O_{i}^{l}$$

where ${B}_{i}^{h}$ is the Error that is being propagated backwards.

### Forward–forward algorithm

The FF algorithm is a greedy multi-layer learning procedure. Rather than doing a forward and a backward pass like in backpropagation, it does two identical forward passes on distinct data with opposing objectives^44^. The positive pass operates on positive/real data and adjusts the weights to increase the goodness (sum of the squared activities) in every hidden layer, i.e., make each layer ‘excited’ about positive data. The negative pass operates on “negative data” (artificially corrupted data) and adjusts the weights to decrease the goodness in every hidden layer, i.e., make each layer less excited about negative data. Each layer has its own loss function and is trained independently of the other. Their interconnection influences overall network performance. A comparative analysis between BP and FF algorithms has been listed in Table 1, which contains the different parameter-wise comparisons, including parameters, output layers, learning process, computational efficiency, biological plausibility, gradient issues, and memory requirements. Each criterion has been analyzed for both FF and BP algorithms.

**Fig. 3:**
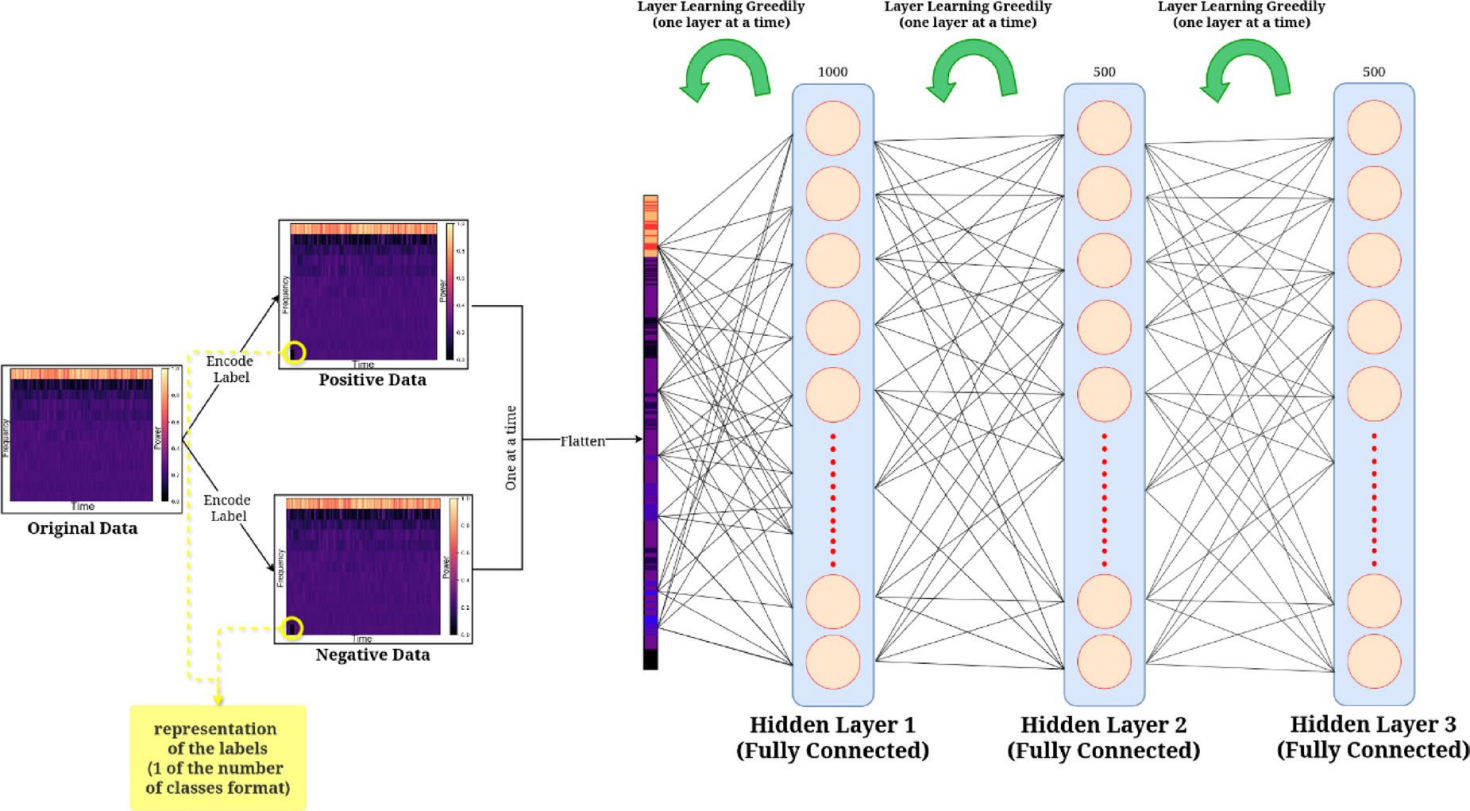
Working of the FF algorithm.

The three major modifications in the FF algorithm over BP which has potential benefits in pest monitoring are: the presence of no dedicated output layer, the presence of local loss functions one for each layer, rather than one global function, learning the representation of the training data so that it can differentiate whether the given input data is positive data or negative data. This approach eliminates the need to store the neural activities or stop to propagate error derivatives. Also, unlike BP, which requires perfect knowledge of the computation performed in the forward pass in order to compute the correct derivatives, forward–forward can be used when the precise details of the forward computation are unknown. These show incredible potential in making use of very low power consumption and computational requirements, which is very crucial in implementing pest control devices in rural areas. However, the FF algorithm has some drawbacks best described by Hinton^44^, that it is somewhat slower than BP and fails to generalize as well on a few of the toy-related issues this work explores, making it unlikely to be used in favor of BP in situations where power is not a concern. Also, since each layer is trained using only the outputs of the previous layer, the lower layers do not receive higher-layer feedback as they do in BP. The working of the FF algorithm has been shown in Fig. 3.

### Label encoding on input data

**Fig. 4:**
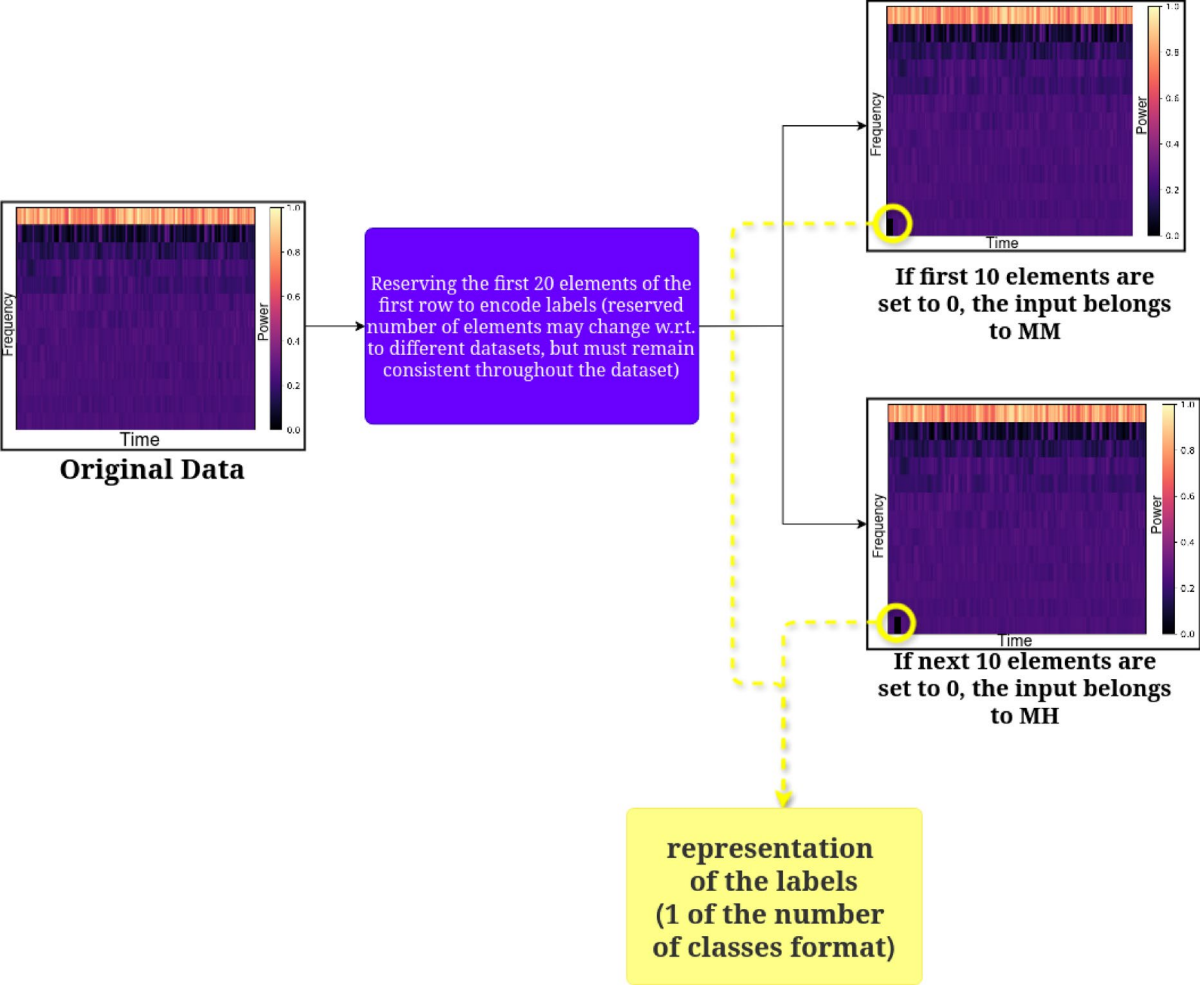
Label encoding on input data.

As in the FF algorithm, the network doesn’t have an output layer, so to achieve supervised learning with FF, labels are included in the inputs themselves. This can be done by embedding one of the representations of the label. The Mel-Frequency Cepstral Coefficients (MFCCs) are one of the popular feature extraction tools used in audio signal processing as they capture the essential frequency characteristics of an audio signal in a form that closely resembles human speech perception^57,58^. They are also used in identifying different kinds of environmental sounds, such as the audio patterns of ships in marine environments^57^. MFCCs are also used in insect sound analysis because they effectively capture the unique acoustic features of different species^59,60^. Similarly, MFCC-based features helped classify bee buzzing sounds at the species level, demonstrating their usefulness in distinguishing insect sounds^60^. For the proposed experiment, the MFCCs of the input data are encoded with the labels (MM and MH). In the proposed implementation, there are two classes, MH (class 0) and MM (class 1). For the positive data generation, if the signal belongs to class 1, the first 5 sample data are set to 0, and the next five are set to 1, and vice versa. Similarly, for negative data generation, if the signal belongs to class 1, the first 5 sample data are set to 1, and the next five are set to 0, and vice-versa. The MFCC parameters are taken as 13 coefficients with a 25-ms window and 50% overlapping. The first 10 columns of the first row were reserved for one-hot label encoding, as shown in Fig. 4. At the first step, the original data is shown, followed by one-hot label encoding as class 0 or 1 at the next step. It was observed during the experimentation stage that the scale of the reservation has a significant impact on the training performance of the network.

### Training of the FF-network

**Fig. 5:**
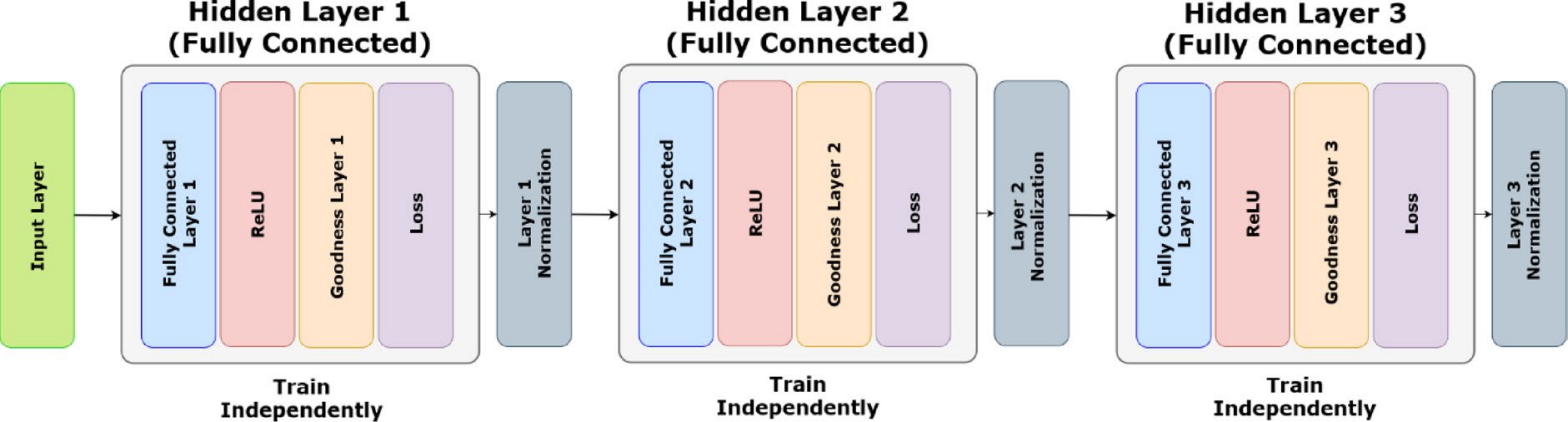
Training architecture of the FF algorithm.

The training of a neural network with the FF Algorithm consists of the following steps. The architecture of the proposed model and the corresponding symbols used in the equations are described in Fig. 5 and Table 2, respectively, followed by a step-wise explanation next. Table 2 contains all the symbols and their corresponding explanations sequentially starting from BP to FF algorithms. It normally works without using backpropagation, rather by running two separate forward passes—one with positive data and another with negative data, allowing each layer to train independently using a goodness measure. The positive and negative data, along with the layers, are shown in Fig. 3.

- During the first of the two forward passes, the positive data is forwarded through a fully connected layer. Here, the input data is the melspectrograms extracted from the input audio signals.
- It is transformed non-linearly by an activation function. For the experiments, ReLU had the best performance as compared to the other activation functions, including sigmoid and tanh. The detection accuracies are found to be less than 91% for both sigmoid and tanh, as compared to the ReLU of 97%. In the original implementation of the FF algorithm also ReLU has also been used as an activation function.
- The goodness of the current hidden layer is calculated. Hinton^44^, proposed the sum of the squared activities in a layer as the goodness function. But there can be many other possibilities. The goodness function proposed in this paper is mentioned in the upcoming section.
- The goodness threshold value is set. It is one of the hyperparameters of the network. The objective of the network is to get the goodness of the positive data well above this threshold and the goodness of the negative data well below it. Choosing the correct range of this threshold value is crucial, as it has a very significant impact on training.
- Loss for the current hidden layer is calculated. The proposed loss function has been discussed in a later section. In theory, each hidden layer can have different loss functions, but during experimentation, the same loss function has been used throughout.
- Using gradient descent, the weights and biases between the previous and the current hidden layer are updated to minimize the loss function.
- During the second forward pass, the negative data is forwarded through the network. It receives the same treatment as the positive data. The only point of difference is the loss function, which is designed in such a way that it decreases with the increase in the goodness for positive data and the decrease in the goodness for negative data.
- After a hidden layer is trained, a layer normalization is performed on that hidden layer.
- So, the input data representation of the previous hidden layer is used to train the next hidden layer. These steps are repeated until all of the hidden layers are trained. The Goodness Function can be expressed as

6 $$G_{h}^{ + } = \sigma \left( {\mathop \sum \limits_{j} \left( {O_{j}^{h} } \right)^{2} - \theta } \right)$$

Here,* θ* is some threshold value. It is a hyperparameter that controls the scale of the gradient. The aim of the learning is to make the goodness well above the threshold value for positive/real data and well below that value for negative/false data. The training Architecture of the FF Algorithm can be expressed as Fig. 5.

### Modified goodness function

Activation functions are pivotal in determining the performance of machine learning models by introducing non-linearity, which enables the modeling of complex data patterns to optimize neural network performance^61^. The impact of different goodness functions on model performance remains an underexplored area in machine learning. Upon experimenting with various potential goodness functions, including Mean Square, Mean Absolute, and RMS, it was observed that the RMS of activities of a layer significantly outperformed Hinton’s proposed goodness function, i.e. sum of squared activities, during training. Traditionally, RMS plays a crucial role in audio signal processing because it measures the average power or loudness, which mimics the hearing pattern of humans. Instead of just looking at peak values, RMS gives a smoother, more consistent sense of the intensity of the audio signal over time, which is very helpful in mixing audio signals^62^. The RMS method evens out quick changes in the sound, making it a reliable way to judge overall energy in the audio^63^. It’s also useful for controlling gain and managing compression, which helps in separating noise from the main audio signal^62^. During the experimentation, it was observed that the RMS of the activation units of a hidden layer performed more than 5% better than the goodness function proposed by Hinton. This finding demonstrates that the choice of goodness function can substantially affect model accuracy and efficiency.

These results highlight the need for further exploration of novel goodness functions. The observed performance variations suggest that undiscovered functions may hold the potential for improving model outcomes. These findings not only contribute to the current body of knowledge but also underscore the importance of expanding research efforts in this domain to unlock new approaches for improving the algorithm. The goodness function is at the forefront of the algorithm. Different goodness functions can have very different impacts. During the experimentation, it was observed that the RMS of the activation units of a hidden layer performed much better than the goodness function proposed by Hinton^44^.

7 $$G_{h}^{ + } = \sigma \left( {\sqrt {\frac{1}{n} \mathop \sum \limits_{j} \left( {O_{j}^{h} } \right)^{2} } - \theta } \right)$$

where *n* is the number of units in layer *h*.

8 $$G_{h}^{ + }>> \theta$$

9 $$G_{h}^{ - } < < \theta$$

where, ${G}_{h}^{+}$ and ${G}_{h}^{-}$ are the probabilities that the input vector is classified as positive and negative, respectively.

### Modified loss function

**Fig. 6:**
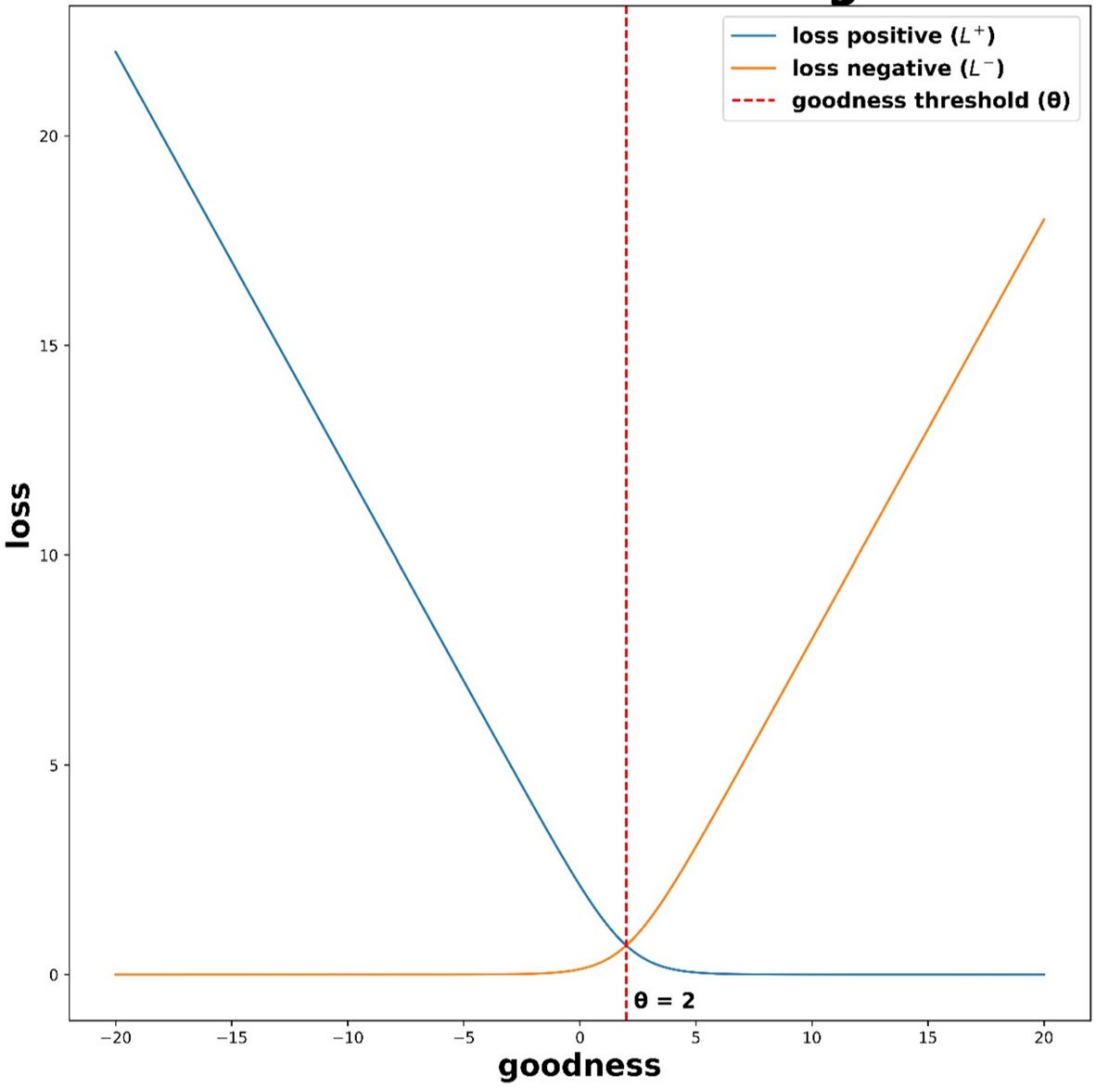
Goodness function comparison.

The Loss Function plays a crucial role in the FF algorithm. The BP algorithm has a global loss function for the entire network. This is essentially why it needs to back-propagate the error to compute the gradients of the loss with respect to the weights. But the forward–forward algorithm has local loss functions, one for each layer. They can be the same or different from each other. The loss function needs to be designed in such a way that it enables each particular layer to have goodness well above the threshold for positive data and well below the threshold for negative data, as shown in Fig. 6, which shows the variation of the goodness function. Since the loss functions in FF do not have access to the labels (correct output) explicitly, they must be creatively designed in such a way that they can effectively differentiate between positive and negative data. It has been observed that squaring the loss function at the end improves the model’s performance. In this paper, the following loss function is proposed and expressed as

10 $$L_{h} = \left( {L_{h}^{ + } + L_{h}^{ - } } \right)^{2}$$

11 $$L_{h}^{ + } = \mathop \sum \limits_{i} \log \left( {1 + e^{{ - \left( {G_{ + } , i - \theta } \right)}} } \right)$$

12 $$L_{h}^{ - } = \mathop \sum \limits_{i} \log \left( {1 + e^{{ - \left( {G_{ - } , i - \theta } \right)}} } \right)$$

13 $${\Delta }W_{ij}^{h} = - \eta \frac{{\partial L_{h} }}{{\partial W_{ij}^{h} }}$$

where, ${L}_{h}^{+}$ and ${L}_{h}^{-}$ are the Loss functions at layer h for positive and negative data, respectively.

### Layer normalization

As explained before, layer normalization is performed on each hidden layer after its training. If layer normalization is not performed, then after training the first hidden layer, the second hidden layer can easily distinguish between positive and negative data just by the length of the activity vector in the first hidden layer. There is no need to learn any new features. Therefore, it is important to perform layer normalization before using it as input to the next hidden layer. This removes all of the information that was used to determine the goodness in the first hidden layer and forces the next hidden layer to use information in the relative activities of the neurons in the first hidden layer. In the first hidden layer, the activity vector has both an orientation and a length. Only the orientation is transferred to the following layer; the length is utilized to define the goodness for that layer^44^.

### Evaluation and testing

**Fig. 7:**
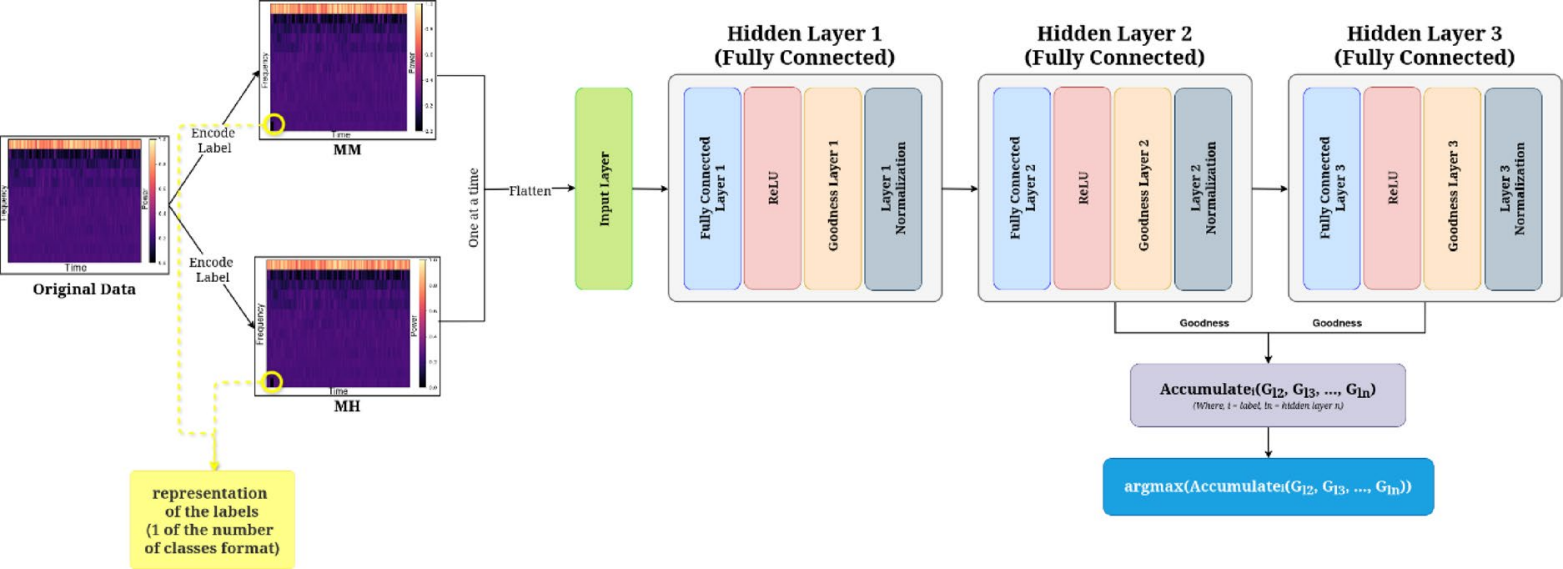
Testing architecture of the FF algorithm.

Just like training, the evaluation of the FF-based network also differs significantly from the evaluation in the BP algorithm. In the proposed implementation, the PyTorch framework has been used as the deep learning framework. The following steps are adopted to evaluate the model through the testing architecture shown in Fig. 7. The Abbreviations used in the manuscript are listed in Table 3.

- Since the true label is unknown, the input data is encoded with each of the distinct labels one at a time.
- The encoded data is passed forward through the network.
- The goodness value for each of the hidden layers is calculated.
- The goodness values of all but the first hidden layer are accumulated.
- After following these steps for each label, the label with the highest accumulated goodness is chosen as the most appropriate one.

**Table 3:** Abbreviation used in the manuscript.

Abbreviations	Details
STE	Short time energy
FF	Forward–forward algorithm
RMS	Root mean square
BP	Back propagation
ASR	Automatic speech recognition
MH	Melolontha hippocastani
MM	Melolontha melolontha
SGD	stochastic gradient descent
KNN	k-nearest neighbor algorithm
SVM	Support vector machine
MFCC	Mel-frequency cepstral coefficients

## Result analysis

The simulation results have been analyzed from the context of the selection of STE level for the specific task of insect detection in soil, along with the effect of goodness threshold values. The proposed model has also been compared with baseline models and another dataset for the scalability analysis in this section. In the end, the effect of environmental sounds has been studied.

### Effect of segmentation & STE level

**Fig. 8:**
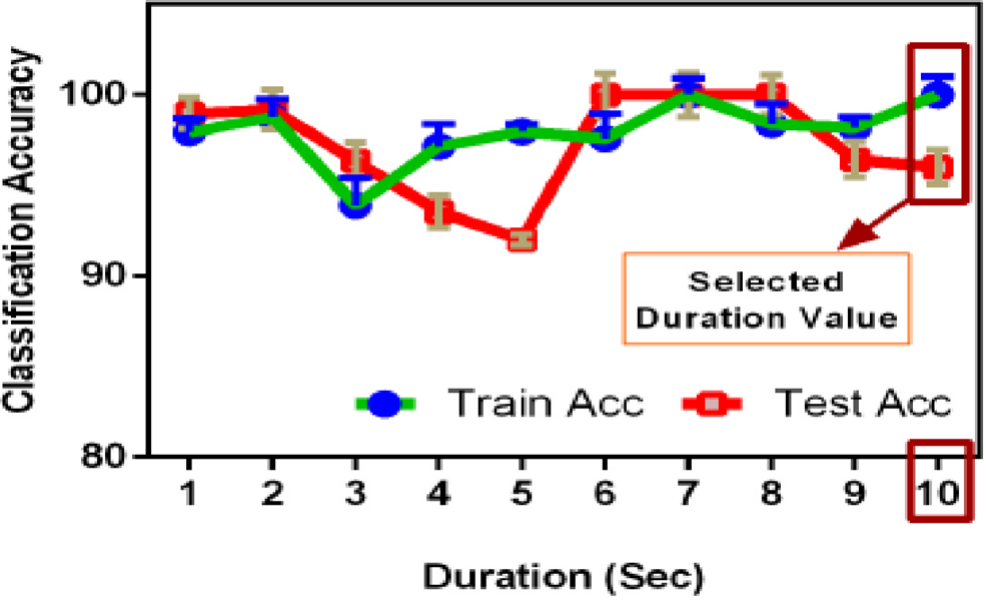
Effect of segmentation on classification accuracy.

**Fig. 9:**
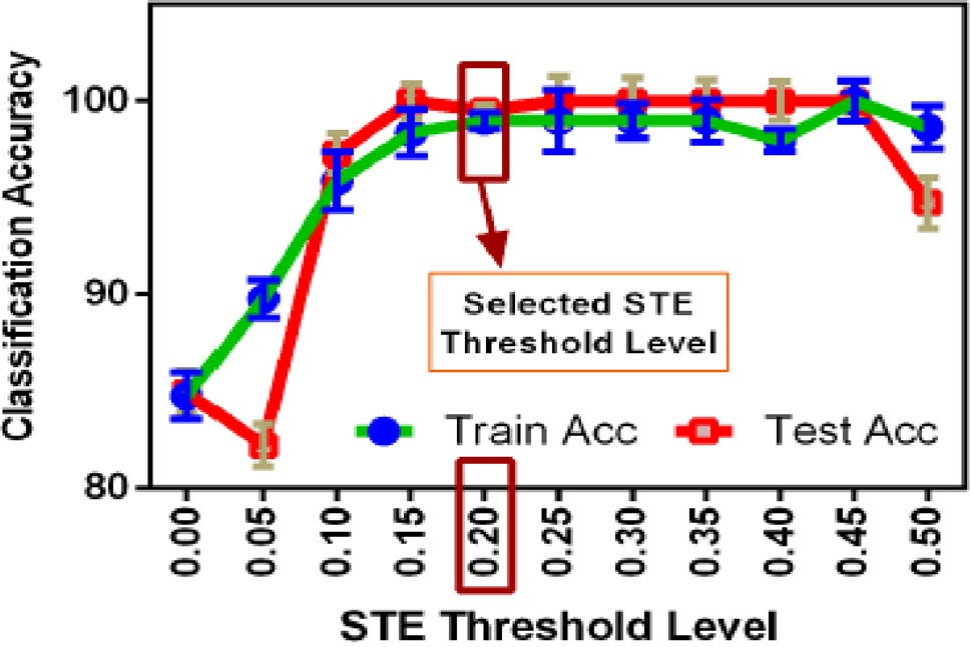
Effect of STE threshold level on classification accuracy.

As the dataset deals with non-speech sounds, the standard voice activity detection for speech signals may not be applied for better performance. In the proposed method, depending on the detection accuracy, a comparative analysis has been performed by changing the duration of segmentation as well as the STE threshold level. The results have been plotted in Fig. 8. The classification accuracies are calculated for both training and testing, and it can be observed that for the duration of ten seconds, the segmentation is working better. Initially, the duration of each audio clip is approximately 5 min, which makes the processing computationally expensive, as well as less accurate in detection. The lowest accuracy has been observed at the segmentation duration of 5 s and improves after that. By using 10-s duration, the training and testing accuracies are satisfactory, and so this duration has been selected for segmentation. After getting the segmentation duration fixed, the audio clips in the dataset have been rearranged with each 10 s duration. In the next step, the framing and windowing have been done for audio preprocessing. For the selection of frames which has relevant audio information, the audio activity detection has been performed by using STE with the proper threshold. A comparative analysis of the selection of the STE threshold level on Classification Accuracy has been done in Fig. 9. It has been observed that with a threshold level of 0.2, the classification accuracy is satisfactory for both training and testing. Hence, in the implementation of the proposed algorithm, a frame size of 25 ms with Hamming windowing along with a 0.2 threshold level has been used.

### Goodness threshold value & number of layers selection

After the selection of the parameters for preprocessing, at the next step, the selection of the threshold value of the Goodness function and the number of layers in the architecture of the modified FF algorithm has been done. The goodness values of all but the first hidden layer are accumulated using the RMS values. The proposed model has been tested in multiple speech recognition datasets, and it has been observed that with three layers (neural units of 500, 1000, and 1000 layers 1, 2, and 3, respectively) performs better than other architectures.

**Fig. 10:**
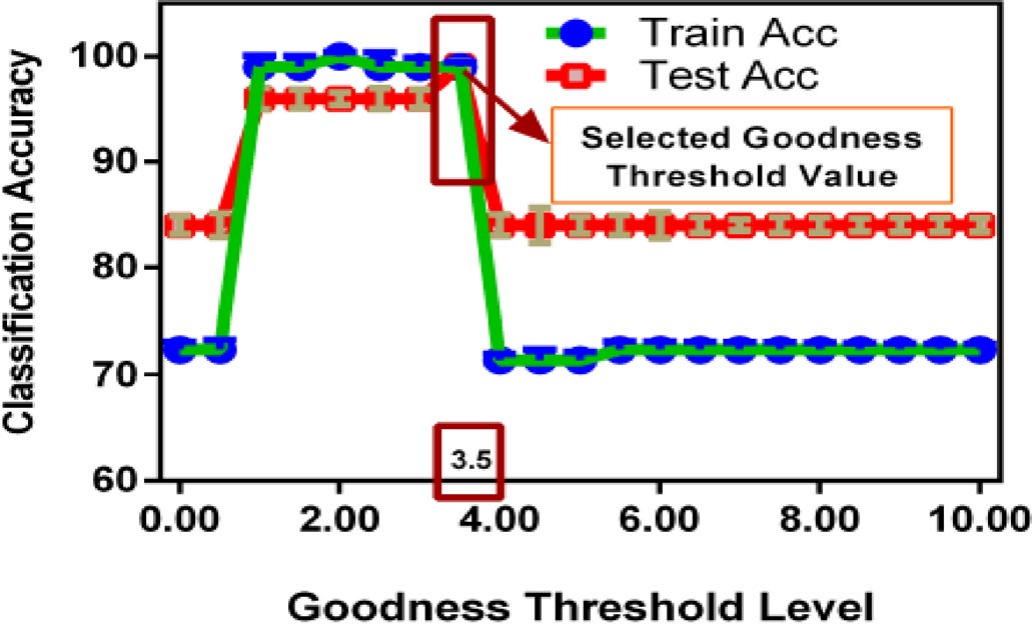
Goodness value selection.

Considering this, in the proposed modified FF model, three layers have been used with 500, 1000, and 1000 neural units. The hyperparameters are taken as 500 epochs, 0.1 learning rate, and 1234 seed value. For the selection of the Goodness threshold value, a comparative analysis has been performed by using training and testing classification accuracies and plotted in Fig. 10. It has been observed that the threshold value of 3.5 is performing better than the other values. Therefore, 3.5 has been selected as the Goodness threshold value for the implementation of the proposed algorithm.

### Comparison with baseline model

For comparative analysis of the proposed model, the performance comparison has been done with five baseline models, which are used for similar audio-related applications, including MFCC with K-NN (Model-1)^18^, MFCC with SVM (Model-2)^64^, MFCC with Random Forest (Model-3)^64^, MFCC with BP (Model-4)^65^, and MFCC with FF (Model-5)^44^. The details of the parameters of these five models are given in Supplementary Data. Simulation has been conducted using Python (version 3.10) within an Anaconda environment. The processing workflow utilized the NumPy (version 1.24) and Librosa (version 0.10) libraries for time–frequency analysis and feature extraction, including the computation of MFCCs. Audio signal processing was performed on a computer equipped with an 11th Generation Intel® Core™ i5-11320H processor (3.20 GHz, 4 cores) and 24 GB of RAM. The simulation results are listed in Table 4, which contains six models to be compared along with their evaluation parameters, including classification accuracy, precision, recall, F-1 Score, and Computational Complexity. K-fold cross-validation helps to get a more accurate sense of how a model will perform on new, unseen data. By testing the model on different parts of the dataset multiple times, it reduces the chance of error in the results analysis^66^. In the proposed implementation, a stratified k-fold cross-validation scheme has been used for training and testing to ensure each class contains the same class distribution in every fold. Here, the value of k has been taken as 5 and the dataset has been randomly divided into five equal subsets, with four subsets used for training and one for testing. Each subset has 280 audio samples, and the process is repeated five times to ensure each subset is used once for testing.

**Table 4:** Performance comparison with baseline models.

Models	Classification accuracy	Precision	Recall	F-1 score	CCP	AUC
Model-1	0.91	0.91	0.90	0.91	0.64	0.93
Model-2	0.93	0.92	0.91	0.91	0.57	0.94
Model-3	0.92	0.92	0.92	0.92	0.56	0.94
Model-4	0.94	0.93	0.92	0.93	0.64	0.95
Model-5	0.85	0.85	0.84	0.84	0.58	0.87
Proposed model	0.97	0.97	0.96	0.97	0.40	0.96

For proper evaluation of the proposed model using STE & RMS-based modified Forward Forward Algorithm (SRFF), classification accuracy, precision, recall, and F-1 score have been used and listed in Table 4. For the analysis of computational complexity, a quantitative evaluation measure called the Computational Complexity parameter (CCP) is used, similar to the computational speed proposed in^67,68^ and Real Time Factor^69^. The benchmark is that the CCP value should be less than 1 for real-time implementation, and the lower the value, the better. The CCP is defined as

14 $$CCP = \frac{{T_{P} \times S_{g} }}{{T_{D} }} \times 100$$

where the processing time of the algorithm is *T*_*P*_ and the total duration of the dataset is *T*_*D,*_ and *S*_*g*_ is the segmentation ratio. The lower value of CCP indicates the lower computational complexity. The Classification Accuracy is calculated from four values: T_+_ (True Positive), F_+_ (False Positive), F_−_ (False Negative), and T_−_ (True Negative). In the present model, T_+_ is the number of MH samples predicted accurately as MH, T_−_ is the number of MM samples predicted accurately as MM, F_+_ is the number of MM samples predicted as MH, and F_−_ is the number of MH samples predicted as MM. The evaluation matrices are defined as

15 $$Classification \;Accuracy = \frac{{\left( {T_{ + } } \right) + \left( {T_{ - } } \right)}}{{\left( {T_{ + } } \right) + \left( {T_{ - } } \right) + \left( {F_{ + } } \right) + \left( {F_{ - } } \right)}}$$

16 $$PR = Precision = \frac{{\left( {T_{ + } } \right)}}{{\left( {T_{ + } } \right) + \left( {F_{ + } } \right)}} \;\;\;RC = Recall = \frac{{\left( {T_{ + } } \right)}}{{\left( {T_{ + } } \right) + \left( {F_{ - } } \right)}}$$

17 $${\text{F}} - 1Score = \frac{{2 \times \left( {PR \times RC} \right)}}{{\left( {PR + RC} \right)}}$$

By comparing the CCP values, it can be observed that the proposed model has lower computational complexity, as due to the STE level-based segmentation selection, the *S*_*g*_ value is comparatively lower than compared to the other models. Similarly, comparing the Classification Accuracy, the ranks of the models in ascending order are Model-5, 1, 3, 2, 4, and the proposed model. In the descending order of the F-1 score, the ranks are as follows: proposed model, Model-4, 3,2,1, and 5. The ranks in the ascending order of Precision and Recall are Model-5, 1, 3, 2, 4, and the proposed model. The Proposed Model has the highest AUC (Area Under the Receiver Operating Characteristic Curve) at 0.96. It ranks positives and negatives very well. This holds true even with a challenging class distribution. On an average level, for most of the evaluation parameters, the proposed model achieves a better average classification accuracy of more than 5% and minimum computational complexity of 20% than the standard baseline models for the insect detection tasks. The computational complexity has been reduced mainly due to enhanced segmentation techniques used in the proposed algorithm. The unnecessary computations have been reduced by only analysing the selected samples of pest sound where the pest activity has been recorded. The initial lengthy recordings have been segmented into smaller-duration recordings, which helped in saving the computations.

**Fig. 11:**
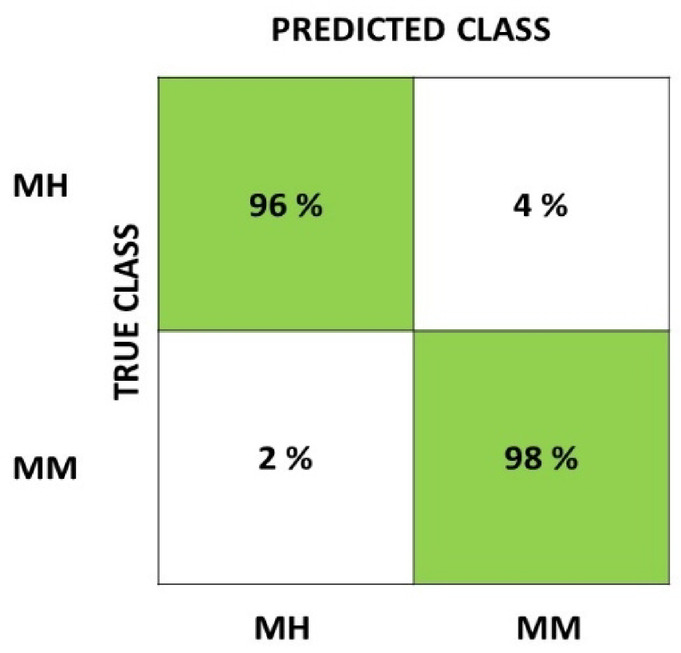
Confusion matrix of the proposed model.

**Fig. 12:**
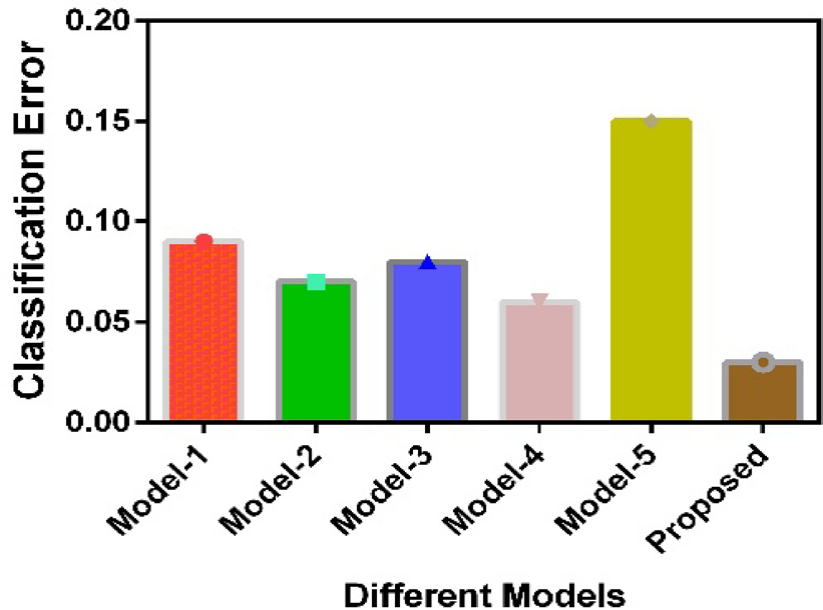
Classification error analysis.

The confusion matrix is shown in Fig. 11, which demonstrates that the proposed model correctly predicts 96% of MH cases and 98% of MM cases, with just 4% of MH labeled as MM and 2% of MM labeled as MH. These low error rates show the model is highly accurate and reliable, with strong recall for both classes. This is especially important in pest detection tasks, where correct predictions are crucial. The balanced performance across both categories also suggests the model isn’t biased toward either class, making it a trustworthy choice for real-world applications. The classification error analysis of the proposed model with the baseline models is shown in Fig. 12. The proposed model stands out with the lowest error, demonstrating that it’s the most accurate of all the models. Model 5, on the other hand, has the highest error and performs the worst. Models 2, 3, and 4 show similar results, while Model 1 performs better than Model 5 but not as well as the other models. These results suggest the proposed model is highly accurate and dependable. This is achieved mainly due to the two basic modifications in the segmentation operation at the preprocessing level and the modified FF approach. In the baseline models, the major challenge lies in segmenting the relevant portions of the data, which creates problems in extracting the relevant features. Even though MFCC features are extracted, the raw input data that has been given to the model to process plays a crucial role, which is reflected in the results. The FF algorithm is also showing improvement in training more accurately than other baseline models with low computational complexity.

To check the scalability, robustness, and generalizability of the proposed model, it has been tested with an insect pest dataset, which is generated from the rice and pulses insect dataset in stored food grains^70^. More than 2000 samples with 48 kHz sampling frequency, with an average duration of 10 s, having two infestation conditions, have been tested, such as high and low, with the proposed FF implementation, with storage noise conditions. It has been observed that the proposed method has demonstrated more than 95% detection accuracy. This shows the better scalability of the proposed model for multiple insect sound-related datasets. In the baseline models, which use the traditional audio-based pest detection methods, the computational requirement is on the higher side, which is one major limitation in the real-time implementation in rural areas. Additionally, the presence of local loss functions, one for each layer in the proposed model, helps to increase the model’s performance in terms of accuracy, whereas in traditional methods, one global loss function is used. With the default loss function, the squared loss is found to be around 87% while with the modified loss function, it increases to 90%.

### Effect of the environmental noise levels

**Fig. 13:**
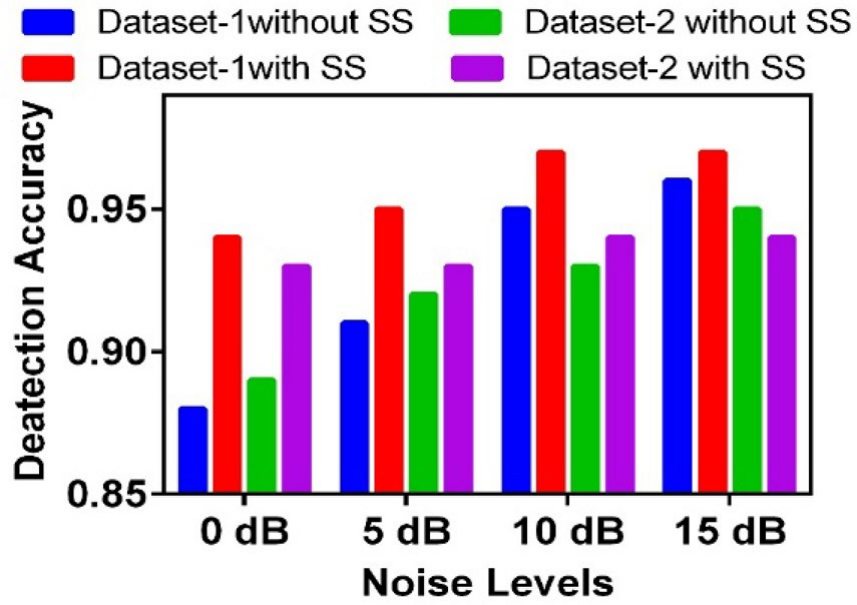
Effect of environmental noise on performance.

Due to the study of soil insects, there is always a chance of surrounding background noise of the environment, including forests. To study the effect of background noise on the proposed model, the two datasets are combined with real-life background environmental noises of the forest at different signal-to-noise ratio (SNR) levels, similar to the Noizeus dataset^37^. SNR is a comparative measure that expresses the strength of the desired signal relative to the background noise, typically in decibels (dB). After the noisy dataset preparation, the classification task is performed using the proposed model. The simulation results are shown in Fig. 13, which contains the effect of noise on classification accuracy at different SNR levels for both datasets. The simulation is performed with the use of an additional simple yet effective low noise reduction algorithm known as Spectral Subtraction (SS)^71^. The SS algorithm works on the noise level estimation and reduction in the frequency domain without affecting the phase of the signal. It can be observed that the effect of background noise can have a significant impact on the performance of the detection algorithm. However, the performance can be improved by using the noise reduction algorithm. Reducing the noise at the low SNR level is challenging compared to the higher SNR values.

Spectral subtraction is a practical and effective method for cleaning up speech signals in noisy environments. It works by estimating the background noise and removing it from the audio, which helps improve clarity without needing to know the exact type of noise in advance. Recent improvements use models that consider how humans actually hear sound, focusing on noise reduction where it’s most noticeable^71^. Other methods adjust to changing noise in real time, preventing issues like unnatural sound artifacts^72^. Some techniques break the audio into frequency bands and clean each one based on its noise level, which helps preserve speech quality. Newer approaches even combine spectral subtraction with machine learning to handle more unpredictable noise scenarios^73^. It has been observed from the literature that spectral subtraction works well on noises that can be separable in the frequency domain. Noises like environmental sounds, engine noises, and crowded place noises have different frequency components as compared to speech and insect sounds^73^. By using the SS algorithm at the first step before using the proposed SRFF algorithm, the performance has been enhanced by a minimum of 4%. At Low SNR conditions, like 0 dB and 5 dB, the detection performance clearly improves when the SS algorithm is used. For example, both datasets show a noticeable jump in accuracy from around 0.88 to up to about 0.93 while using the SS algorithm. This means the SS algorithm really helps when the noise is high and the signals are hard to detect. Even at 5 dB, where conditions are a bit better, the algorithm still gives a boost, especially for Dataset-1. Overall, the SS algorithm makes a real difference in keeping detection reliable when the noise level is challenging, which is crucial for practical applications in tough environments.

Deploying audio-based soil pest detection in real farms means dealing with real-world challenges like noise, weather, power, and data. Microphones need to be tuned to pick up pest sounds in the 100–1000 Hz range while ignoring wind or machinery noise^74^. The sensors must be tough—ideally waterproof and dust-resistant (IP67)—to survive underground conditions. Since each sensor can generate up to 1 GB of audio data daily, local processing or compression is key to keeping storage and transmission practical^75^. Power is also a concern; low-energy chips (under 100 mW) and small solar panels (5–10 W) can keep the system running off-grid. These practical tweaks are essential for making the technology work reliably in the field. Audio-based soil detection is a promising but costly setup, with installation plus system setup may be between $5000–$10,000. For maintenance, the cost will be approximately $100 per setup. Audio-based soil detection can make farming more sustainable by helping farmers catch issues like pests or soil stress early, just by listening to what’s happening underground. Instead of spraying pesticides across entire fields, they can treat only the areas that really need it, cutting down on chemicals and protecting helpful insects and soil life. It can also give insights into root growth or soil activity, helping farmers water and manage their land more efficiently, which is better for the environment and long-term soil health. This technology could be useful in farming communities around the world, including in developing countries, because it provides a simple way to keep an eye on soil health and detect pests early^76,77^. That said, upfront costs, access to electricity and internet, and the need for technical know-how might make it harder to use in places with fewer resources. Making the system affordable, easy to maintain, and able to work without constant connectivity would be important to help farmers everywhere benefit from it.

The audio-based soil detection system can integrate with other smart farming technologies like automated irrigation and crop monitoring. It provides real-time insights into soil and root health, helping adjust watering schedules and complementing above-ground monitoring tools. Together, these technologies offer farmers a more complete picture of their crops, enabling earlier problem detection and smarter resource management. To speed up training for pest detection models, especially with large or complex datasets, several smart strategies can be adopted. Acoustic analysis helps smart farming by detecting hidden pests early. This allows farmers to act before visible damage occurs, and they support healthier crops with smart agriculture. Starting with pre-trained models and fine-tuning them (transfer learning) saves time, while using lightweight or efficient architectures keeps things fast without sacrificing accuracy. Instead of using deep learning models, application-specific audio features can be prepared to reduce computational time. Training can be made quicker with batch processing and the use of GPUs. Pre-processing audio into formats like spectrograms ahead of time also helps. Instead of collecting tons of new data, using data augmentation and focusing on harder or less common cases can make training more effective and efficient. AI-driven pest detection using microphones and audio processing raises ethical issues, including potential privacy breaches from unintended recordings, ambiguity around data ownership, and the diminishing role of traditional knowledge. Ensuring ethical use requires transparent data policies and accountability.

## Conclusion

This paper presents a new approach to soil pest detection using speech recognition through a modified Forward–forward algorithm and improved segmentation techniques. By using the STE-based segmentation, the unnecessary processing has been reduced while still accurately identifying pest sounds underground. Unlike traditional backpropagation models, this approach uses application-specific goodness and loss functions designed for soil pest sound. This makes the model more effective in noisy, real-world conditions. The proposed model has consistently outperformed the baseline models by achieving higher accuracy even in low SNR conditions with reduced computational complexity. In the future, this method has the potential to be used beyond pest detection. This method can be used in farming to monitor animals by analyzing the signs of stress or illness through audio signal analysis. It can also help spot pests, detect leaks in irrigation systems, and check on machine health monitoring through audio analysis. Even the plant stress conditions can be monitored for stress using appropriate sensors. In the future, the concept of transfer learning from animal sound datasets, along with active learning and implementations on training on mobile edge devices, can be explored. In addition to the audio analysis, a multi-modal fusion approach can be done, including audio and video analysis. Overall, these future applications can help to develop a smart farming practice in agriculture.

## Supplementary Information

Supplementary Information.

## Data Availability

The datasets analyzed during the current study are available in the Dryad Digital Repository. (10.5061/dryad.2j87692)^51,78^. Görres, Carolyn-Monika; Chesmore, David^78^. Data from: Active sound production of scarab beetle larvae opens up new possibilities for species-specific pest monitoring in soils [Dataset]. Dryad. 10.5061/dryad.2j87692 The insect dataset can be accessed from the Data Hub IIT Kharagpur (https://www.datahubkgp.org)^70^. The code has been uploaded at https://github.com/TKDSP/srff No animals were harmed during data collection.

## References

[1] Lima, M. C. F., de Almeida Leandro, M. E. D., Valero, C., Coronel, L. C. P. & Bazzo, C. O. G. Automatic detection and monitoring of insect pests—A review. *Agriculture* **10**, 161. 10.3390/agriculture10050161 (2020).

[2] Wu, Q., Zeng, J. & Wu, K. Research and application of crop pest monitoring and early warning technology in China. *Front. Agric. Sci. Eng.* **9**, 19–36. 10.15302/J-FASE-2021411 (2022).

[3] Redlich, S., Martin, E. A. & Steffan-Dewenter, I. Sustainable landscape, soil and crop management practices enhance biodiversity and yield in conventional cereal systems. *J. Appl. Ecol.* **58**(4), 740–752. 10.1111/1365-2664.13821 (2021).

[4] Samreen, T. et al. Sensing techniques in precision agriculture for pest and disease management. *Environ. Sci. Proc.* **23**(1), 16. 10.3390/environsciproc2022023016 (2022).

[5] Dhanaraj, R. K., Ali, M. A., Sharma, A. K. & Nayyar, A. Deep multibranch fusion residual network and IoT-based pest detection system using sound analytics in large agricultural field. *Multimed. Tools Appl.* **83**, 40215–40252. 10.1007/s11042-023-16897-3 (2024).

[6] Sharma, R. P., Ramesh, D., Pal, P., Tripathi, S. & Kumar, C. IoT-enabled IEEE 802.15. 4 WSN monitoring infrastructuredriven fuzzy-logic-based crop pest prediction. *IEEE Internet Things J.* **9**, 3037–3045. 10.1109/JIOT.2021.3094198 (2021).

[7] Ali, M. A., Dhanaraj, R. K. & Nayyar, A. A high-performance, AI-enabled IoT pest detection system using sound analytics in large agricultural fields. *Microprocess. Microsyst.* **103**, 104946. 10.1016/j.patcog.2023.109071 (2023).

[8] Adedeji, A. A. et al. Non-destructive technologies for detecting insect infestation in fruits and vegetables under postharvest conditions: A critical review. *Foods* **9**(7), 927. 10.3390/foods9070927 (2020). 32674380 10.3390/foods9070927 PMC7404779

[9] Kaur, A., Agarwal, M. & Saini, R. Speech endpoint detection using short-time energy and CNN–BiLSTM. *Int. J. Inf. Technol.* **15**, 2767–2775. 10.1007/s41870-023-01466-6 (2023).

[10] Branding, J., von Hörsten, D., Böckmann, E., Wegener, J. K. & Hartung, E. InsectSound1000 an insect sound dataset for deep learning based acoustic insect recognition. *Sci. Data* **11**, 475. 10.1038/s41597-024-03301-4 (2024). 38724595 10.1038/s41597-024-03301-4 PMC11082239

[11] Mankin, R. W., Hagstrum, D. W., Smith, M. T., Roda, A. L. & Kairo, M. T. Perspective and promise: A century of insect acoustic detection and monitoring. *Am. Entomol.* **57**, 30–44. 10.1093/ae/57.1.30 (2011).

[12] Phung, Q. V., Ahmad, I., Habibi, D. & Hinckley, S. Automated insect detection using acoustic features based on sound generated from insect activities. *Acoust. Aust.* **45**, 445–451. 10.1007/s40857-017-0095-6 (2017).

[13] Mankin, R. W. Applications of acoustics in insect pest management. *CABI Review*10.1079/PAVSNNR20127001 (2012).

[14] Gao, Y. et al. Application of machine learning in automatic image identification of insects—A review. *Ecol. Inform.* **79**, 102539. 10.1016/j.ecoinf.2024.102539 (2024).

[15] Muppala, C. & Guruviah, V. Machine vision detection of pests, diseases, and weeds: A review. *J. Phytol.* **12**, 9–19. 10.25081/jp.2020.v12.6145 (2020).

[16] Dhanaraj, R. K. & Ali, M. A. Deep learning-enabled pest detection system using sound analytics in the internet of agricultural things. *Eng. Proc.* **58**, 123. 10.3390/ecsa-10-16205 (2023).

[17] Wang, Z. et al. Detection of insect-damaged maize seed using hyperspectral imaging and a hybrid 1D-CNN-BiLSTM model. *Infrared Phys. Technol.* **137**, 105208. 10.1016/j.infrared.2024.105208 (2024).

[18] Shetty, M. V. & Kumar, Y. D. S. *Audio-Based Classification of Insect Species Using Machine Learning Models: Cicada, Beetle, Termite, and Cricket*. *arXiv preprint* arXiv:2502.13893 (2025). 10.48550/arXiv.2502.13893

[19] Chen S. et al. *Deep Learning-Based Acoustic Mosquito Detection in Noisy Conditions*. *arXiv preprint* arXiv:2207.13843 (2022). 10.48550/arXiv.2207.13843

[20] Aman, E. & Wang, H.-C. A deep learning-based embedded system for pest bird sound detection and proximity estimation. *Eur. J. Eng. Technol. Res.* **9**(1), 53–59. 10.24018/ejeng.2024.9.1.3150 (2024).

[21] Liu, S. Y. Artificial intelligence (AI) in agriculture. *IT Pro.* **22**(3), 14–15. 10.1109/MITP.2020.2986121 (2020).

[22] Rotz, S. et al. Automated pastures and the digital divide: How agricultural technologies are shaping labour and rural communities. *J. Rural Stud.* **68**, 112–122. 10.1016/j.jrurstud.2019.01.023 (2019).

[23] Trippa, G. et al. Next-generation methods for early disease detection in crops. *Pest Manag. Sci.* **80**(1), 123–135. 10.1002/ps.7733 (2024). 10.1002/ps.7733 37599270

[24] Sishodia, R. P., Ray, R. L. & Singh, S. K. Applications of remote sensing in precision agriculture: A review. *Remote Sens.* **12**(19), 3136. 10.3390/rs12193136 (2020).

[25] Liu, J. & Wang, X. A multimodal framework for pepper diseases and pests detection. *Sci. Rep.* **14**, 28973. 10.1038/s41598-024-80675-w (2024). 39578611 10.1038/s41598-024-80675-w PMC11584720

[26] Gibb, R., Browning, E., Glover-Kapfer, P. & Jones, K. E. Emerging opportunities and challenges for passive acoustics in ecological assessment and monitoring. *Methods Ecol. Evol.* **10**, 169–185. 10.1111/2041-210X.13101 (2019).

[27] Mennill, D. J., Burt, J. M., Fristrup, K. M. & Vehrencamp, S. L. Accuracy of an acoustic location system for monitoring the position of duetting songbirds in tropical forest. *J. Acoust. Soc. Am.* **119**, 2832–2839. 10.1121/1.2184988 (2006). 16708941 10.1121/1.2184988 PMC2247711

[28] Wright, D., Hammond, N., Thomas, G., MacLeod, B. & Abbott, L. K. The provision of pest and disease information using information communication tools (ICT); an Australian example. *Crop Prot.* **103**, 20–29. 10.1016/j.cropro.2017.08.023 (2018).

[29] Liu, D. et al. Detection of forestry pests based on improved YOLOv5 and transfer learning. *Forests* **14**(7), 1484. 10.3390/f14071484 (2023).

[30] Zhang J., Liu Z. & Yu K. MSFNet-CPD: *Multi-Scale Cross-Modal Fusion Network for Crop Pest Detection*. *arXiv preprint* arXiv:2505.02441 (2025). 10.48550/arXiv.2505.02441

[31] Duan J., Ding H. & Kim S. *A Multimodal Approach for Advanced Pest Detection and Classification*. *arXiv preprint* arXiv:2312.10948 (2023). 10.48550/arXiv.2312.10948

[32] Saki, M. et al. *Precision Soil Quality Analysis Using Transformer-Based Data Fusion Strategies: A Systematic Review*. *arXiv preprint* arXiv:2410.18353 (2024). 10.48550/arXiv.2410.18353

[33] Legner C. M., Tylka G. L. & Pandey S. *Robotic Agricultural Instrument for Automated Extraction of Nematode Cysts and Eggs From Soil to Improve Integrated Pest Management*. *arXiv preprint* arXiv:2205.11757 (2022). 10.48550/arXiv.2205.11757 10.1038/s41598-021-82261-w PMC7864952 33547348

[34] Dai, M., Dorjoy, M. M. H., Miao, H. & Zhang, S. A new pest detection method based on improved YOLOv5m. *Insects* **14**, 54. 10.3390/insects14010054 (2023). 36661982 10.3390/insects14010054 PMC9863093

[35] Zhang, Y. & Lv, C. TinySegformer: A lightweight visual segmentation model for real-time agricultural pest detection. *Comput. Electron. Agric.* **218**, 108740. 10.1016/j.compag.2024.108740 (2024).

[36] Faiß, M. & Stowell, D. Adaptive representations of sound for automatic insect recognition. *PLoS Comput. Biol.* **19**, e1011541. 10.1371/journal.pcbi.1011541 (2023). 37792895 10.1371/journal.pcbi.1011541 PMC10578591

[37] Hu, Y. & Loizou, P. C. Subjective evaluation and comparison of speech enhancement algorithms. *Speech Commun.* **49**, 588–601. 10.1016/j.specom.2006.12.006 (2007). 18046463 10.1016/j.specom.2006.12.006 PMC2098693

[38] Priya, R. & Ramesh, D. ML based sustainable precision agriculture: A future generation perspective. *Sustain. Comput. Inform. Syst.* **28**, 100439. 10.1016/j.suscom.2020.100439 (2020).

[39] Srivastava, N., Hinton, G., Krizhevsky, A., Sutskever, I. & Salakhutdinov, R. Dropout: A simple way to prevent neural networks from overfitting. *J. Mach. Learn. Res.* **15**, 1929–1958. 10.5555/2627435.2670313 (2014).

[40] Goodfellow, I., Bengio, Y. & Courville, A. *Deep Learning* (MIT Press, 2016). 10.1007/s10710-017-9314-z.

[41] Hastie, T., Tibshirani, R., Friedman, J. H. & Friedman, J. H. *The Elements of Statistical Learning: Data Mining, Inference, and Prediction* Vol. 2 (Springer, 2009). 10.1007/978-0-387-84858-7.

[42] Holt, J. L. & Hwang, J. N. Finite precision error analysis of neural network hardware implementations. *IEEE Trans. Comput.* **42**, 281–290. 10.1109/12.210171 (1993).

[43] LeCun, Y., Bengio, Y. & Hinton, G. Deep learning. *Nature* **521**, 436–444. 10.1038/nature14539 (2015). 26017442 10.1038/nature14539

[44] Hinton, G. The forward–forward algorithm: *Some Preliminary Investigations*. *arXiv preprint* arXiv:2212.13345 (2022). 10.48550/arXiv.2212.13345

[45] Lillicrap, T. P., Santoro, A., Marris, L., Akerman, C. J. & Hinton, G. Backpropagation and the brain. *Nat. Rev. Neurosci.* **21**, 335–346. 10.1038/s41583-020-0277-3 (2020). 32303713 10.1038/s41583-020-0277-3

[46] Gautham, S. R., Nair, S., Jamadagni, S., Khurana, M. & Assadi, M. Exploring the feasibility of Forward forward algorithm in neural networks. *In: 2024 International Conference on Advances in Modern Age Technologies for Health and Engineering Science (AMATHE),* pp. 1–6 (2024). 10.1109/AMATHE61652.2024.10582053

[47] Chen, X., Liu, D., Laydevant, J. & Grollier, J. Self-contrastive forward–forward algorithm. *Nat. Commun.* **16**(1), 5978. 10.1038/s41467-025-61037-0 (2025). 40595637 10.1038/s41467-025-61037-0 PMC12217723

[48] Barua, P. D. et al. Innovative fibromyalgia detection approach based on quantum-inspired 3LBP feature extractor using ECG signal. *IEEE Access* **11**, 101359–101372. 10.1109/ACCESS.2023.3315149 (2023).

[49] Karimi, A., Kalhor, A. & Tabrizi, M. S. Forward layer-wise learning of convolutional neural networks through separation index maximizing. *Sci. Rep.* **14**, 8576. 10.1038/s41598-024-59176-3 (2024). 38615041 10.1038/s41598-024-59176-3 PMC11385581

[50] Ram, B. G., Oduor, P., Igathinathane, C., Howatt, K. & Sun, X. A systematic review of hyperspectral imaging in precision agriculture: Analysis of its current state and future prospects. *Comput. Electron. Agric.* **222**, 109037. 10.1016/j.compag.2024.109037 (2024).

[51] Görres, C.-M. & Chesmore, D. Active sound production of scarab beetle larvae opens up new possibilities for species specific pest monitoring in soils. *Sci. Rep.* **9**, 10115. 10.1038/s41598-019-46121-y (2019). 31300666 10.1038/s41598-019-46121-y PMC6626128

[52] Ahmed, A. & Lawaye, A. CNN-based speech segments endpoints detection framework using short-time signal energy features. *Int. J. Inf. Technol.* **15**, 2791–2799. 10.1007/s41870-023-01466-6 (2023).

[53] Loizou, P. C. *Speech Enhancement: Theory and Practice* 2nd edn. (CRC Press, 2013). 10.1201/b14529.

[54] Moattar, M. H. & Homayounpour, M. M. A simple but efficient real-time voice activity detection algorithm. In: 2009 *17th European Signal Processing Conference*, pp. 2549–2553 (2009). https://ieeexplore.ieee.org/abstract/document/7077834

[55] Cilimkovic, M. *Neural Networks and Back Propagation Algorithm*. *Institute of Technology Blanchardstown, Blanchardstown Road North Dublin* Vol. 15 (2015). https://www.academia.edu/download/51924347/NeuralNetworks.pdf

[56] Sharma, S., Sharma, S. & Anidhya, A. Understanding activation functions in neural networks. *Int. J. Eng. Appl. Sci. Technol.* **4**, 310–316 (2020).

[57] Dixit, V., Rao, K. S. & Manjunath, D. Improved spectral dynamic features extracted from audio data for classification of marine vessels. *Intell. Mar. Technol. Syst.* **1**, 1–10. 10.1007/s44295-024-00029-0 (2016).

[58] Povey, D. et al. The Kaldi Speech Recognition Toolkit. *In: Proc. IEEE Workshop on Automatic Speech Recognition and Understanding* (ASRU 2011), pp. 1–4 (2011). http://publications.idiap.ch/downloads/papers/2012/Povey_ASRU2011_2011.pdf

[59] Wei, J.-Q., Wang, X.-Y., Zheng, X.-L. & Tong, X. Stridulatory organs and sound recognition of three species of longhorn beetles (Coleoptera: Cerambycidae). *Insects* **15**(11), 849. 10.3390/insects15110849 (2024). 39590448 10.3390/insects15110849 PMC11594338

[60] Ratnayake, A. M. B., Yasin, H. M., Naim, A. G. & Abas, P. E. Buzzing through data: Advancing bee species identification with machine learning. *Appl. Syst. Innov.* **7**, 62. 10.3390/asi7040062 (2024).

[61] Jagtap, A. D. & Karniadakis, G. E. How important are activation functions in regression and classification? A survey, performance comparison, and future directions. *J. Mach. Learn. Model. Comput.* **4**(1), 21–75. 10.1615/JMachLearnModelComput.2023047367 (2023).

[62] Haykin, S. & Van Veen, B. *Signals and Systems* 2nd edn. (Wiley, 2003).

[63] Hastie, T., Tibshirani, R. & Friedman, J. *The Elements of Statistical Learning* 2nd edn. (Springer, 2009). 10.1007/978-0-387-84858-7.

[64] Silva, D. F., Souza, V. M. D., Batista, G. E., Keogh, E. & Ellis, D. P. Applying machine learning and audio analysis techniques to insect recognition in intelligent traps. In: 2013 *12th International Conference on Machine Learning and Applications* vol. 1,pp. 99–104 (2013). 10.1109/ICMLA.2013.24

[65] Mankin, R., Hagstrum, D., Guo, M., Eliopoulos, P. & Njoroge, A. Automated applications of acoustics for stored product insect detection, monitoring, and management. *Insects* **12**, 259. 10.3390/insects12030259 (2021). 33808747 10.3390/insects12030259 PMC8003406

[66] Barhoush, M., Hallawa, A. & Schmeink, A. Speaker identification and localization using shuffled MFCC features and deep learning. *Int. J. Speech Technol.* **26**, 185–196. 10.1007/s10772-023-10023-2 (2023).

[67] Bhowmick, A. & Chandra, M. Speech enhancement using voiced speech probability based wavelet decomposition. *Comput. Electr. Eng.* **62**, 706–718 (2017).

[68] Bhowmick, A., Chandra, M. & Biswas, A. Speech enhancement using Teager energy operated ERB-like perceptual wavelet packet decomposition. *Int. J. Speech Technol.* **20**(4), 813–827. 10.1016/j.compeleceng.2017.01.013 (2017).

[69] Wang, Y.-H., Yeh, C.-H., Young, H.-W.V., Hu, K. & Lo, M.-T. On the computational complexity of the empirical mode decomposition algorithm. *Phys. A Stat. Mech. Appl.* **400**, 159–167. 10.1016/j.physa.2014.01.020 (2014).

[70] AI for Interdisciplinary Cyber-Physical Systems (AI4ICPS). *Environmental AI Projects*. https://www.ai4icps.in/projects?section=ai_env (Accessed 14 Aug 2024).

[71] Boll, S. F. Suppression of acoustic noise in speech using spectral subtraction. *IEEE Trans. Acoust. Speech Signal Process.* **27**(2), 113–120. 10.1109/TASSP.1979.1163209 (1979).

[72] Karam, M., Khazaal, H. F., Aglan, H. & Cole, C. Noise removal in speech processing using spectral subtraction. *J. Signal Inf. Process.* **5**(2), 32–41. 10.4236/jsip.2014.52006 (2014).

[73] Cohen, I. Noise spectrum estimation in adverse environments: improved minima controlled recursive averaging. *IEEE Trans. Speech Audio Process.* **11**(5), 466–475. 10.1109/TSA.2003.811544 (2003).

[74] Kadyrov, D., Sutin, A., Sedunov, N., Sedunov, A. & Salloum, H. Vibro-acoustic signatures of various insects in stored products. *Sensors* **24**, 6736. 10.3390/s24206736 (2024). 39460216 10.3390/s24206736 PMC11511369

[75] Baumüller, H. The little we know: An exploratory literature review on the utility of mobile phone-enabled services for smallholder farmers. *J. Int. Dev.* **30**(1), 134–154. 10.1002/jid.3314 (2018).

[76] Paddock, K. J. et al. Soil microbes from conservation agriculture systems reduce growth of Bt-resistant western corn rootworm larvae. *J. Pest Sci.* **97**, 1677–1689. 10.1007/s10340-023-01725-2 (2024).

[77] Kalyani, Y. & Collier, R. A systematic survey on the role of cloud, fog, and edge computing combination in smart agriculture. *Sensors* **21**(17), 5922. 10.3390/s21175922 (2021). 34502813 10.3390/s21175922 PMC8434609

[78] Görres, C.-M. & Chesmore, D. Data from: Active sound production of scarab beetle larvae opens up new possibilities for species-specific pest monitoring in soils. Dryad 10.5061/dryad.2j87692 (2019). 10.1038/s41598-019-46121-y PMC6626128 31300666

