# Supplementary material for: Audio signal analysis using a modified forward–forward algorithm with enhanced segmentation for soil pest detection
Source: Sci Rep. 2025 Aug 27;15:31542. doi: 10.1038/s41598-025-15770-7 (PMC12391374; doi:10.1038/s41598-025-15770-7)
Supplement: Supplementary file 1 — Supplementary Information. [file 41598_2025_15770_MOESM1_ESM.pdf]

Supplementary Data S1

Squared loss with ablation study results in supplementary

With the default loss function, the squared loss is found to be around 87 % while with the modified loss function, it increases to 90% .

Supplementary Data S2

Model Architectures

1. Model-1 – 13-dimensional MFCC Features with nearest neighbors (k) as 5 in KNN model [41]
2. Model-2 - 13-dimensional MFCC Features with Linear SVM and gamma value is auto selected [42]
3. Model-3 - 13-dimensional MFCC Features with default parameters of Random Forest and depth = 8 [42]
4. Model-4 - 13-dimensional MFCC Features with Backpropagation architecture (1000, 500, 500), and learning rate = 0.001, num of epochs = 200 [43]
5. Model-5 - 13-dimensional MFCC Features with default FF [28]

Supplementary Data S3

t-SNE Plot for dataset-2 with MFCC Features

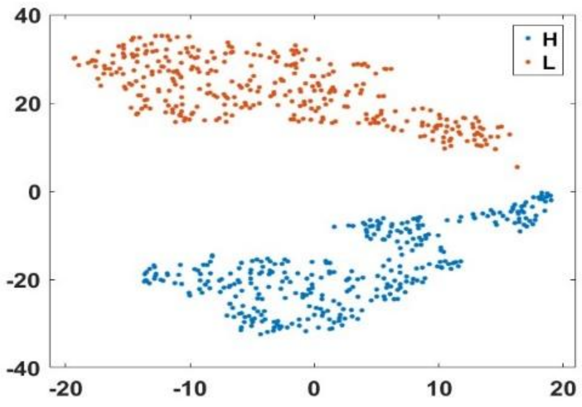

Supplementary Data S4

original vs. segmented waveforms

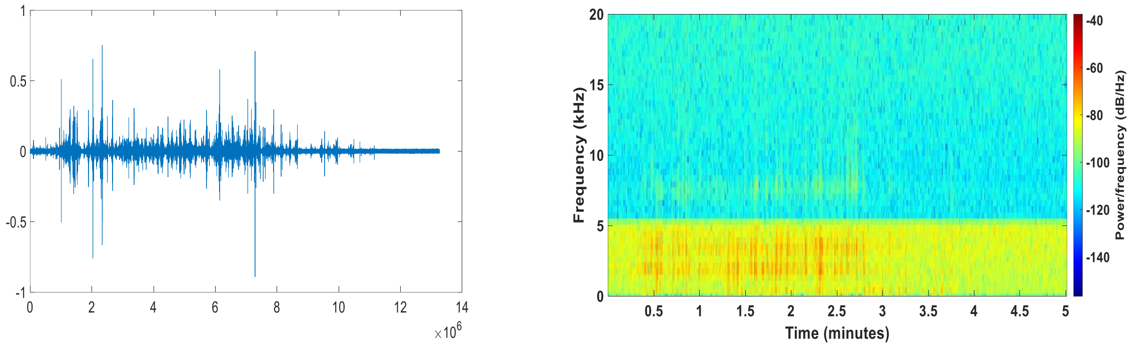

Original Signal Waveforms & Spectrograms

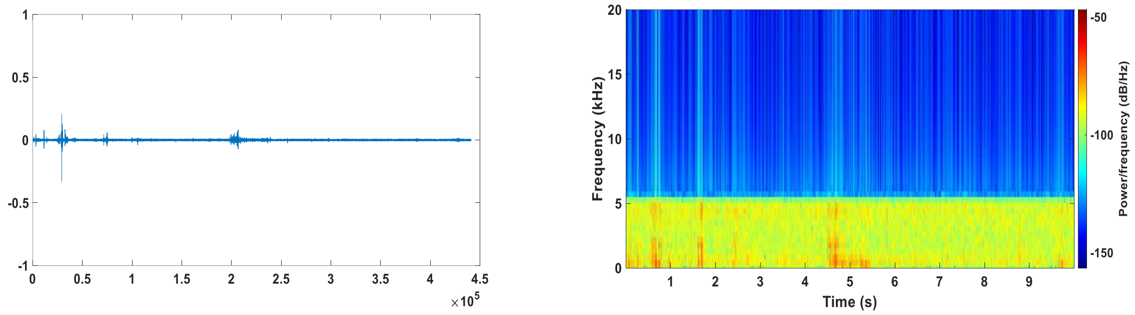

Segmented Signal Waveforms & Spectrograms
